# Assessment of bacterial profile, antimicrobial susceptibility status, and associated factors of isolates among hospitalized patients at Dessie Comprehensive Specialized Hospital, Northeast Ethiopia

**DOI:** 10.1186/s12866-024-03224-5

**Published:** 2024-04-04

**Authors:** Assefa Sisay, Abdurahaman Seid, Selamyhun Tadesse, Wagaw Abebe, Agumas Shibabaw

**Affiliations:** 1 Department of Medical Laboratory Science, College of Health Science, Woldia University, Woldia, Ethiopia; 2 Department of Medical Laboratory Science, College of Medicine and Health Science, Wollo University, Dessie, Ethiopia

**Keywords:** Bacterial profile, Antimicrobial susceptibility profile, Hospitalized patients, Dessie

## Abstract

**Background:**

Antimicrobial resistant bacteria among hospitalized patients are becoming a major public health threat worldwide, mainly in developing countries. Infections by these multidrug resistant pathogens cause high rate of mortality, prolong hospital stays, and affect individual and country economies in greater amounts. Thus, this study aimed to assess the bacterial profile, antimicrobial susceptibility status, and associated factors of isolates from hospitalized patients at the Dessie Comprehensive Specialized Hospital.

**Methodology:**

This hospital-based cross-sectional study was conducted between February and April 2021. Consecutive sampling was used to select the study participants. All bacterial isolates were identified using standard bacteriological techniques. Antibiotic susceptibility testing was performed using disk diffusion technique. The data was analyzed using SPSS version 25. Descriptive statistics and logistic regression were used. A P-value of less than 0.05 was considered statistically significant.

**Results:**

Of 384 clinical samples (blood, urine, stool, wound, vaginal discharge, and ear discharge) processed 180 (46.9%) were culture positive. Overall, *Escherichia coli* was the predominant isolate (41; 22.8%), followed by *Staphylococcus aureus* (36; 20%). Most of the isolates were from blood (70; 38.9%). The level of overall drug resistance of the gram-negative bacteria isolates for ampicillin, tetracycline, and cotrimoxazole was (104; 88.1%), (79; 75.9%), and (78; 75.0%), respectively. The overall multidrug rate of isolates was 143 (79.4%). Variables such as history of invasive procedures, chronic underlying diseases, history of hospitalization, and habit of eating raw animal products were statistically significant for the acquisition of bacterial infection.

**Conclusions and recommendation:**

*E. Coli* and *S. aureus* were the most common isolates. Most of the isolates were resistant to commonly prescribed antibiotics. And also, consumption of raw animal products, chronic underlying disease, previous hospitalization, history of invasive procedures, and educational status were associated with the acquisition of bacterial infections. Therefore, routine antimicrobial susceptibility testing, proper patient management, wise use of antibiotics in clinical settings and health education are recommended.

## Introduction

The global distribution and growing number of pathogens resistant to antimicrobial drugs are potentially one of the greatest threats to global health, especially in sub-Saharan Africa, where the burden of infectious diseases is high. According to recent estimates, antimicrobial resistant (AMR) infections cause approximately 700,000 deaths per year, with the number expected to rise to 10 million by 2050 if current trends continue [1, 2].

Blood stream, urinary tract, wound, and lower respiratory tract infections are the most common illnesses caused by both gram-positive and gram-negative bacteria [3–8]. The most common pathogens associated with bacterial infections in both health care and community settings are *Escherichia coli* (*E. coli*), *Staphylococcus aureus* (*S. aureus*), *Klebsiella pneumonia* (*K. pneumonia*), *Pseudomonas aeruginosa* (*P. aeruginosa*), *Streptococcus pneumoniae* (*S. pneumonia*), and *Salmonella* spp [5, 9].

Microbial diseases cause 25% of the world’s 60 million yearly fatalities. Despite great improvements in infection control methods, infections caused by drug-resistant organisms remain a major source of morbidity and mortality in hospitalized patients and the community, especially in sub-Saharan African countries [5, 6]. Populations with low immunity, such as pediatrics, pregnant women, and patients with chronic illnesses who frequently visit health facilities, have a high incidence of bacterial infections, which increase mortality and morbidity [6, 10].

A recent report estimated that 10 million deaths will be attributed to AMR by 2050, and 100 trillion dollars of the world’s economic output will be lost if substantive efforts are not made to address this threat [2, 11]. Sub-Saharan Africa, including Ethiopia, has few comprehensive antimicrobial surveillance strategies and scarce infection prevention and control programs [1, 5, 6]. Unwise antimicrobial use and poor guidelines for over-the-counter sales of antibiotics are increasing in these countries, increasing the emergence and spread of multidrug resistant (MDR) pathogens [1, 5, 11].

Even if much data is required for the intervention of AMR at the national level, there is insufficient information showing the country-wide prevalence of the bacteria, antimicrobial susceptibility results, and MDR profiles. Furthermore, due to insufficient human resources, laboratory facilities, and diagnostic kits for antimicrobial susceptibility testing, data are scarce in hospitals. Therefore, this study aimed to identify the bacterial profiles, antimicrobial susceptibility patterns, and associated factors among hospital admitted patients at Dessie Comprehensive Specialized Hospital, Northeast Ethiopia.

## Methods

### Study area

The study was conducted at the Dessie Comprehensive Specialized Hospital. Dessie Comprehensive Specialized Hospital is a referral hospital that provides healthcare services for more than four million people in the area. It has more than 700 client loads per day (source: Dessie Comprehensive Specialized Hospital Report, 2020). The hospital consists of six major wards: emergency, surgical, medical, obstetrics and gynecology, pediatric, neonatal intensive care unit (NICU), and adult intensive care.

### Study design, period, and population

A hospital-based cross-sectional study was conducted among hospitalized patients at the Dessie Comprehensive Specialized Hospital from February to April 2021. Patients admitted to the medical, surgical, obstetrics, gynecology, pediatric, and intensive care unit (ICU) wards for less than 48 h and who had clinical evidence of bacterial infection were considered the study population.

### Sample size and sampling technique

A single population proportion formula was used to calculate the sample size, assuming a 95% confidence level, 5% margin of error, and taking 50% prevalence of community acquired bacterial infections in hospitalized patients. The final sample size was 384. The study participants were consecutively included until the required sample size was achieved.

### Operational definition

Hospitalized patients: Patients admitted to the medical, surgical, obstetrics, gynecology, pediatric, and intensive care unit (ICU) wards for less than 48 h and who had clinical evidence of bacterial infection.

### Data collection

Information on demographic variables and clinical data was collected from each participant through face-to-face interviews using a structured questionnaire and by reviewing patients’ medical records in consultation with their respective physicians. All the data were collected and recorded using a trained data collector.

### Sample collection and processing

Clinical samples, including blood, urine, stool, wound, vaginal discharge, and ear discharge, were collected based on the type of disease which progressed in the participants. Samples were collected during the study period, transported, and processed according to standard procedures [12].

#### Blood sample

Venous blood (10, 2, and 1 mL from adults, children, and neonates, respectively) was aseptically collected. Immediately after collection, blood samples were added to tryptic soy broth (TSB) (Oxoid, Ltd.) in two bottles for each patient at approximately 1 h intervals [13] and incubated at 37 ^o^C. If growth was observed, subculturing was performed on MacConkey agar (MAC) (HiMedia, India) and blood agar (BA) (HiMedia, India) plates. For chocolate agar (HiMedia, India) it was incubated in a 5–10% CO_2_ atmosphere using a candle jar at 37 °C for 24 to 48 h. When no visible growth was detected, the TSB tubes were incubated for 7 days before being reported as negative.

#### Urine sample

Ten milliliters of freshly voided midstream urine were collected using a sterile, wide-mouthed, and leak-proof container. For patients with a urinary catheter, the aperture was cleaned in a circular motion from the aperture opening outward using a cleansing solution. The open end of the catheter was placed in the specimen container, and 5–10 mL of urine was dripped into the container. Using a calibrated wire loop, 1 µL (0.001 mL) of a well-mixed urine sample was inoculated into a cysteine lactose electrolyte-deficient medium (CLED) (HiMedia, India) and incubated for 24 h. Colonies were counted to check for the presence of significant growth, and those yielding bacterial growth of ≥ 10^5^ CFU/mL were considered significant bacteriuria [12]. The colonies from CLED agar were subcultured in MAC (Oxoid, Ltd.) and BA (Oxoid, Ltd.) and incubated at 37 °C for 24 h.

#### Wound sample

Wound swab/pus aspirate samples from each participant were collected aseptically from the depth of the wound with cotton swabs dipped in normal saline using the Levine technique. The sample was immersed in a container of brain-heart infusion transport medium. The brain heart infusion culture was incubated for 24 h, sub-cultured on MAC agar and BA plates, and re-incubated for 24 h at 37 ^o^C.

#### Sputum sample

Two milliliters of sputum samples were collected from patients suspected of having a bacterial lower respiratory tract infection using sterile, clean, wide-necked, leak-proof containers and transported within 2 h [13]. Gram staining was performed on purulent portions of each sputum sample. Sputum samples were examined to determine the appropriateness of the culture. Samples with fewer than 10 polymorphonuclear neutrophils per epithelial cell were considered contaminated by saliva and rejected. Those sputum specimens of good quality were inoculated with MAC, BA, and chocolate (5–10% CO_2_) and incubated for 24–48 h at 35–37 °C [13].

#### Stools sample

Two grams of fecal specimen were collected, and rectal swabs were collected from children that could not provide a stool. The sample was transported using Cary-Blair transport medium (Oxoid, Basingstoke, UK), inoculated onto MAC, and incubated at 35–37 °C for 24 h. The sample was cultured on xylose lysine deoxycholate (XLD) agar from patients suspected of *salmonella* and *Shigella spp.* infections.

#### Cerebrospinal fluid sample (CSF)

Two to five milliliters of CSF were collected aseptically using two tubes by an experienced professional between the fourth and fifth lumbar vertebrae of the arachnoid space and were transported immediately. One tube was centrifuged for Gram staining, and the other tube was used for culturing [13]. For cloudy specimens, Gram staining was performed before centrifugation; if not cloudy, CSF was centrifuged for Gram staining. After centrifugation, the sediment was cultured on MAC, BA, and chocolate (5–10% CO_2_) and incubated for 24–48 h at 35–37 °C [12].

#### Vaginal and ear discharges sample

Vaginal and ear discharges were collected aseptically using sterile cotton swabs and transported using tryptic soya broth media. Vaginal and ear discharge was inoculated into MAC, BA, and chocolate (5–10% CO_2_) and incubated for 24–48 h at 35–37 °C [12].

All isolates were preliminarily screened by colony morphology, pigment production (pink to colorless flat or mucoid colonies), and Gram staining techniques. Further identification of the isolates was performed based on relevant biochemical tests [12]. Gram-negative bacteria were identified based on indole production, H2S production on KIA agar, citrate utilization, urease tests, carbohydrate fermentation on KIA, motility tests, and LDC tests. Gram-positive bacteria were identified using the catalase, coagulase, novobiocin, and bacitracin sensitivity tests.

### Antimicrobial susceptibility testing

The antimicrobial susceptibilities of the isolates were determined according to the criteria of the Clinical and Laboratory Standard Institute 2020 (CLSI) using the Kirby-Bauer disk diffusion method on Mueller-Hinton Agar [14]. Antimicrobial disks used for susceptibility testing were cefoxitin (30 µg), clindamycin (2 µg), erythromycin (15 µg), and penicillin G (10 IU). Ampicillin (10 µg), cefotaxime (30 µg), ciprofloxacin (5 µg), chloramphenicol (30 µg), trimethoprim-sulfamethoxazole (1.25/23.25 µg), tetracycline (30 µg), and gentamicin (10 µg) were used for both gram-negative and gram-positive bacteria, whereas ceftriaxone (30 µg) and amoxicillin-clavulanic acid (20/10 µg) were used for gram-negative bacteria. The inoculated plates with antimicrobial disks were incubated at 35–37 ^0^C for 16–18 h, and the diameters of the zones of inhibition were measured using a ruler and interpreted according to CLSI standards [15]. Bacterial isolates that are resistant to three or more antibiotics from different classes are considered multi-drug resistant [16].

### Data and laboratory quality control

The questionnaires were checked for completeness during and after data collection. All laboratory assays were performed while maintaining quality control procedures. The sterility of the medium was assessed by incubating 5% of the batch at 35–37 ^0^C overnight. Antimicrobial sensitivity testing disks were checked using standardized reference strains of *S. aureus* ATCC25923, *E. coli* ATCC25922, and *P. aeruginosa* ATCC27853 [15].

### Data analysis

Data were analyzed using the Statistical Package for Social Sciences (SPSS) version 25. Descriptive statistics, such as frequency and percentage, were calculated and are presented in the tables. Binary logistic regression was used to assess the association between individual explanatory variables and dependent variables. Odds ratios were calculated using the respective confidence intervals. Furthermore, variables with P *≤* 0.2 in the bivariable analysis were entered into multivariable logistic regression. A P-value less than 0.05 was considered to be statistically significant.

## Result

### Socio-demographic and clinical characteristics

Among the 384 study participants enrolled in the study, 230 (59.9%) were male. The age of the study participants ranged from 4 days to 85 years, with a median age of 36 years. The majority of the participants were rural residents (238; 62%) (Table 1).

**Table 1:** Socio demographic and clinical characteristics of the study participants (N = 384) at Dessie Comprehensive Specialised Hospital, Northeast Ethiopia from February to April 2021

Demographic Characteristics	Category	Frequency, n (%)
Age in years	≤ 14	55 (14.3)
	15–29	60 (15.6)
	30–44	155 (40.4)
	45–59	84 (21.9)
	≥ 60	30 (7.8)
Sex	Male	230 (59.9)
	Female	154 (40.1)
Residence	Urban	146 (38.0)
	Rural	238 (62.0)
Educational status	Can’t read and write	100 (26.0)
	Read and write	99 (25.8)
	Primary school	57 (14.8)
	Secondary school	38 (9.9)
	Diploma or certificate	65 (16.9)
	Degree and above	25 (6.5)
Chronic underlying disease	Yes	97 (25.3)
	No	287 (74.7)
Urinary catheterization	Yes	69 (18.0)
	No	315 (82.0)
History of invasive procedure	Yes	45 (11.7)
	No	339 (88.3)

### Bacterial isolation rate

A single specimen was collected from each participant, and the overall culture positivity rate was 180 (46.9%). Among the isolates, gram negative bacteria were predominant (119/180; 66.1%) and from all bacteria *E. coli* (41; 22.8%) was the most prevalent, followed by *S. aureus* (36; 20.0%), and *K. pneumoniae* (21; 11.7%). The majority of the isolates were from blood samples (70; 38.9%) and urine samples (68; 37.8%) (Table 2).

**Table 2:** Proportion of bacterial isolated from different clinical samples (n = 180) among patients admitted for less than 48 h at Dessie Comprehensive Specialized Hospital Northeast Ethiopia from February to April 2021

Isolated spp	Type of specimen collected							
	Blood n (%)	Urine n (%)	Stool n (%)	Sputum n (%)	Wound n (%)	Vaginal dis n (%)	Ear dis n (%)	Total = 180 n (%)
*E.coli*	7 (17.1)	24 (58.5)	5 (12.2)	–	3 (7.3)	–	2 (4.9)	41 (22.8)
*K. pneumonia*	4 (19)	6 (28.6)	–	9 (42.9)	1 (4.8)	–	1 (4.8)	21 (11.7)
*Pseudomonas spp*	7 (46.7)	3 (20)	–	–	5 (33.3)	–	–	15 (8.2)
*Citrobacter spp.*	5 (50)	1 (10)	–	–	4 (40)	–	–	10 (5.5)
*K. oxytoca*	3 (42.9)	1 (14.3)	–	1 (14.3)	2 (28.6)	–	–	7 (3.9)
*C. diversus*	2 (40)	2 (40)	–	–	1 (20)	–	–	5 (2.8)
*E. aerogens*	3 (60)	1 (20)	–	–	1 (20)	–	–	5 (2.8)
*p. mirabilis*	–	4 (80)	–	–	1 (20)	–	–	5 (2.8)
*P. vulgaris*	1 (20)	3 (60)	–	–	1 (20)	–	–	5 (2.8)
*K. ozaenae*	3 (100)	–	–	–	–	–	–	3 (1.7)
Acinetobacter spp.	1 (100)	–	–	–	–	–	–	1 (0.5)
Salmonella spp	–	–	1 (100)	–	–	–	–	1 (0.5)
**Total Gram negative**	**36**	**45**	**6**	**10**	**19**		**3**	**119 (66.1)**
*S. aureus*	18 (50)	14 (389)	–	–	2 (5.6)	1 (2.8)	1 (2.8)	36 (20.0)
*S. saprophyticus*	10 (62.5)	6 (37.5)	–	–	–	–	–	16 (8.9)
*S. epidermidis*	5 (62.5)	3 (37.5)	–	–	–	–	–	8 (4.4)
*S. agalactiae*	1 (100)	–	–	–	–	–	–	1 (0.5)
**Total Gram positive**	**34 (48.6)**	**23 (33.8)**	**0 (0)**	**0 (0)**	**2 (9.5)**	**1 (100)**	**1** (25)	**61 (33.9)**
**Overall**	**70 (38.9)**	**68(37.8)**	**6(3.3)**	**10 (5.5)**	**21(11.7)**	**1 (0.6)**	**4 (2.2)**	**180 (100)**

### Antimicrobial resistance profile of gram negative bacterial isolates

The overall resistance rates of gram negative bacteria to ampicillin, tetracycline, and trimethoprim-sulfamethoxazole were highest at 88.1%, 75.9%, and 75%, respectively. However, these isolates exhibited the lowest overall resistance to ceftriaxone (41.2%). From the isolates *E. coli* showed the lowest resistance to ceftriaxone (39%) but the highest for ampicillin (95.1%) and trimethoprim-sulfamethoxazole (87.8%). The level of resistance of *K. pneumoniae* was highest for ampicillin (81.0%) and tetracycline (76.2%) (Table 3).

**Table 3:** Antimicrobial resistance rate result of gram negative bacteria among patients admitted for less than 48 h to Dessie Comprehensive Specialized Hospital, Northeast Ethiopia, February–April 2021

Isolated spp (n = 119)	Antibacterial agents								
	AMC n (%)	CIP n (%)	CHF n (%)	CN n (%)	SXT n (%)	CRO n (%)	AMP n (%)	CEF n (%)	TET n (%)
*E. coli (41)*	19 (46.3)	18 (43.9)	21 (51.2)	20 (48.8)	36 (87.8)	16 (39)	39 (95.1)	18 (43.9)	31 (75.6)
*K. pneumoniae (21)*	14 (66.7)	9 (42.9)	8 (38.1)	11 (52.4)	14 (66.7)	12 (57.1)	17 (81.0)	11 (52.4)	16 (76.2)
*Pseudomonas spp (15)*	–	9 (60)	–	9 (60)	–	8 (53.3)	14 (93.3)	9 (60)	–
*Citrobacter spp. (10)*	5 (50)	4 (40)	7 (70)	2 (20)	7 (70)	3 (30)	8 (80)	5 (50)	7 (70)
*K. oxytoca (7)*	3 (42.9)	4 (57.1)	3 (42.9)	3 (42.9)	4 (57.1)	2 (28.6)	7 (100)	4 (57.1)	4 (57.1)
*C. diversus (5)*	3 (60)	1 (20)	3 (60)	1 (2)	3 (60)	1 (20)	5 (100)	2 (40)	4 (80)
*E. aerogen s (5)*	3 (60)	2 (40)	2 (40)	2 (40)	3 (60)	2 (40)	3 (60)	2 (40)	3 (60)
*P. mirabilis (5)*	3 (60)	3 (60)	3 (60)	1 [20]	4 (80)	1 [20]	4 (80)	4 (80)	5 (100)
*P.vulgaris (5)*	2 (40)	3 (60)	3 (60)	2 (40)	3 (60)	2 (40)	3 (60)	3 (60)	5 (100)
*K. ozaenae (3)*	2 (66.7)	2 (66.7)	1 (33.3)	2 (66.7)	2 (66.7)	1 (33.3)	3 (100)	2 (66.7)	2 (66.7)
Other gram negative (2)	50 (50)	1 (50)	1 (50)	0 (0)	2 (100)	1 (50)	1 (50)	1 (50)	2 (100)
Overall resistance (119)	55 (53.4)	56 (47.1)	52 (50.5)	53 (44.5)	78 (75.0)	49 (41.2)	104 (88.1)	61 (51.3)	79 (75.9)

CIP, Ciprofloxacin; AMC, AmoxicillinClavulanic acid; CRO, Ceftriaxon; CN, Gentamicin; SXT, Trimethoprim-Sulphamethoxazole; AMP, Ampicillin; CEF, Cefotaxime; CHF, Chloramphenicol; TET, Tetracycline

### Antimicrobial resistance patterns of gram positive bacterial isolates

The overall resistance rates of the gram-positive bacteria to ampicillin, tetracycline, and trimethoprim-sulfamethoxazole were 77.0%, 72.1%, and 71.7%, respectively. *Staphylococcus aureus* showed high resistance to ampicillin (80.6%), penicillin G, tetracycline (72.2% each), and trimethoprim–sulfamethoxazole (69.4%), whereas *S. saprophyticus* showed equal resistance to ampicillin and trimethoprim–sulfamethoxazole (75%) (Table 4).

**Table 4:** Antimicrobial resistance profile of gram positive bacteria among patients admitted for less than 48 h to Dessie Comprehensive Specialized Hospital, Northeast Ethiopia, February–April 2021

Isolated spp (n = 61)		Antibacterial agents									
	FOX n (%)	CL n (%)	E n (%)	P n (%)	CIP n (%)	CHF n (%)	CN n (%)	SXT n (%)	AMP n (%)	CEF n (%)	TET n (%)
*S. aureus (36)*	15 (41.7)	21 (58.3)	15 (41.7)	26 (72.2)	9 (25)	11 (30.6)	8 (22.2)	25 (69.4)	29 (80.6)	16 (44.4)	26 (72.2)
*S. saprophytcus (16)*	–	10 (62.5)	8 (50)	10 (62.5)	6 (37.5)	7 (43.8)	5 (31.3)	12 (75)	12 (75)	5 (31.3)	11 (68.7)
*S. epidermidis (8)*	–	4 (50)	4 (50)	6 (75)	3 (37.5)	4 (50)	3 (37.5)	6 (75)	5 (62.5)	5 (62.5)	6 (75)
*S. agalactiae (1)*	–	0(0)	1 (100)	1 (100)	0(0)	0(0)	–	–	1 (100)	1 (100)	1 (100)
Overall resistance (61)	15 (41.7)	35 (57.4)	28 (45.9)	43 (70.5)	18 (29.5)	22 (36.1)	16 (26.7)	43 (71.7)	47 (77)	27 (44.3)	44 (72.1)

FOX, cefoxitin; CL, clindamycin; CIP, ciprofloxacin; CN, entamicin; E, erythromycin; SXT, trimethoprim-sulfamethoxazole; P, penicillin; AMP, ampicillin; CHF, chloramphenicol; CEF, cefotaxime; TET, tetracycline

### Multidrug resistance profiles of isolates

Of all bacterial isolates, 95% of the isolates were resistant to at least one of the selected antibiotics. The overall multidrug resistance rate (bacteria resistant to three or more different classes of drugs) of isolated bacteria was 143 (79.4%). Among gram negative bacteria a higher MDR rate was shown to *K. pneumoniae* and *E. coli* (90.5% and 90.2%, respectively). Among the gram-positive bacteria, the MDR rates of *S. aureus* and *S. saprophyticus* were 78.8% and 75%, respectively (Table 5).

**Table 5:** Multidrug resistance profile of bacteria isolated among patients admitted for less than 48 h at Dessie Comprehensive Specialized Hospital, Northeast Ethiopia, February–April 2021

	Isolated spp (n = 180)														
*Antibiogram*	*E. coli*	*K. pneumonia*	*S. aureus*	*S. Saprophyticus*	*Pseudomonas*	*Citrobacter spp*	*S. epidermidis*	*K. oxytoca*	*C. diversus*	*E. aerogens*	*P. mirabilis*	*P. vulgaris*	*K. ozaenae*	*Other gram negative*	*S. agalactiae*
R0 (%)	0 (0)	1 (4.8)	3 (8.3)	4 (25)	0 (0)	1 (10)	0 (0)	0 (0)	0 (0)	0 (0)	0 (0)	0 (0)	0 (0)	0 (0)	0 (0)
R1 (%)	1 (2.4)	0 (0)	3 (8.3)	0 (0)	1 (6.7)	0 (0)	0 (0)	1 (14.3)	0 (0)	1 (20)	0 (0)	0 (0)	0 (0)	0 (0)	0 (0)
R2 (%)	1 (2.4)	0 (0)	1 (2.8)	0 (0)	3 (20.0)	0 (0)	3 (37.5)	1 (14.3)	1 (20)	1 (20)	0 (0)	2 (40)	0 (0)	0 (0)	0 (0)
R3 (%)	3 (7.3)	1 (4.8)	3 (8.3)	0 (0)	5 (33.3)	1 (10)	0 (0)	1 (14.3)	0 (0)	0 (0)	0 (0)	0 (0)	1 (33.3)	0 (0)	0 (0)
R4 (%)	3 (7.3)	4 (19)	3 (8.3)	1 (6.3)	3 (20)	1 (10)	0 (0)	0 (0)	1 (20)	00 (0)	1 (20)	0 (0)	0 (0)	0 (0)	1 (100)
R5 (%)	11 (26.8)	4 (19)	2 (5.6)	0 (0)	3 (20)	4 (40)	0 (0)	0 (0)	1 (20)	1 (20)	2 (40)	0 (0)	0 (0)	2 (100)	0 (0)
R6 (%)	15 (36.6)	4 (19)	2 (5.6)	1 (6.3)	0 (0)	2 (20)	1 (12.5)	0 (0)	2 (40)	1 (20)	1 (20)	0 (0)	0 (0)	0 (0)	0 (0)
R7 (%)	7 (17.1)	7 (33.4)	19 (52.8)	10 (62.5)	0 (0)	1 (10)	4 (50)	4 (57.1)	0 (0)	1 (20)	1 (20)	3 (60)	2 (66.7)	0 (0)	0 (0)
MDR	90.2%	90.5%	78.8%	75%	66.7%	80%	62.5%	57.1%	80%	60%	100%	60%	66.67%	100%	100%
Overall MDR	143 (79.4%)														

R0, susceptible to all tested antibiotics; R1–6, resistance to 1, 2, 3, 4, 5, and 6 antibiotics; R7 for ≥ 7 antibiotics. ≥R3: resistance to three or more antibiotics from different classes

### Associated factors of bacterial infection

Bivariable logistic regression analysis was performed for the sociodemographic and clinical variables. Those with *P* ≤ 0.2 were further analyzed using multivariable logistic regression. In the multivariate analysis, clients who couldn’t read and write had higher odds of having bacterial infection than those who had a degree (AOR = 3.84; 95% CI = 1.83–8.03). Participants who had chronic underlying disease were 1.83 times more likely to develop bacterial infection (95% CI = 1.05–3.17) as compared with those who did not. Moreover, history of having an invasive procedure had 2.09 times the odds of acquiring bacterial infection than their counterparts (95% CI = 1.06–4.49) (Table 6).

**Table 6:** Bivariable and multivariable analysis of associated factors for acquiring bacterial infection among study participants attending (N = 384) at Dessie Comprehensive Specialized Hospital from February to April 2021

Characteristics (Total No.)		Bacterial infection		COR (CI 95%)	P-value	AOR	P-value
		Positive N, %	Negative N, %				
Residence	Urban (146)	55(37.7)	91(62.3)	Ref			
	Rural (238)	125(52.5)	113(47.5)	1.83(1.20–2.78)	0.005	0.88(0.378–2.05)	0.77
Educational status	Can’t read and write (100)	32 (32)	68(68)	1.49(0.543–4.09)	0.439	3.84(1.83–8.03)	< 0.001^*^
	Read and write(99)	67(67.1)	32(32.3)	6.63(2.42–18.20)	0.001	2.45(1.03–5.82)	0.043^*^
	Primary school(57)	30(52.6)	27(47.4)	3.52(1.23–10.10)	0.019	1.19(0.37–3.8)	0.78
	Secondary school 38)	18(47.4)	20(52.6)	2.85(0.93–8.57)	0.066	1.32(0.32–5.50)	0.699
	Diploma (65)	27(41.5)	38(58.5)	2.25(0.79–6.37)	0.127	0.57(0.10–3.46)	0.56
	Degree (25)	6 (24)	19(76)	Ref			
Eating raw animal product	Yes (245)	137(55.9)	108(44.1)	2.83(1.83–4.40)	0.001	2.09(1.23–3.55)	0.006^*^
	No (139)	43(30.9)	96(69.1)	Ref			
Previous hospitalization	Yes (77)	47(61)	30 (39)	2.05(1.23–3.42)	0.006	1.96(1.09–3.54)	0.024^*^
	No (307)	133(43.3)	174(56.7)	Ref			
Invasive procedure	Yes (45)	26(57.8)	19(42.2)	1.64(0.88–3.08)	0.121	2.18(1.06–4.49)	0.035*
	No (339)	154(45.4)	185(54.6)	Ref			
chronic disease	Yes (97)	57(58.8)	40(41.2)	1.90(1.19–3.03)	0.007	1.83(1.05–3.17)	0.033*
	No (287)	123(42.9%)	164(57.1%)	Ref			

*Note* COR: crude odds ratio, AOR: adjusted odds ratio, CI: confidence interval, Ref: Reference

*Those have association with bacterial infection

## Discussion

Our study provides a comprehensive description of the epidemiology and microbiological causes of community-acquired infections in a specialized hospital in Northeast Ethiopia. In this study, the prevalence of bacterial infection was 46.9%. This was consistent with studies conducted in Debre Markos, Ethiopia, at 46.7% and 48.7% [17, 18], Bahir Dar, Ethiopia, at 49.4% [19], and Tanzania, at 42.2% [20]. However, this was higher than findings from studies conducted in Hawassa, Ethiopia, at 28% [21] and Tanzania, at 14.8% [22]. Lower findings were observed when compared with studies from Ethiopia, at 64% [23] and Pakistan, at 87.17% [24]. This variation may be due to geographic differences, seasonal variations, differences in study methods, and variations in infection control programs.

In the current study, gram-negative bacteria were the dominant isolates, accounting for 66.1%. This was supported by studies conducted in Ethiopia (67.8%) [21] and Indonesia (68.6%) [25]. The most predominant gram-negative isolate in this study was *E. coli (*22.8%), followed by *K. pneumoniae* (11.7%), which is similar to other studies conducted in Ethiopia [26, 27] and Uganda [28]. This might be due to their major abundance as a normal flora and the fact that they can easily be transported to sterile areas of the body and cause infection [29].

In contrast, 33.9% of the isolates were gram-positive bacteria. Among these, *S. aureus* was the leading isolate. This finding is supported by studies conducted in Gondar, Ethiopia [27], and Indonesia [25]. Overall, *E. coli* and *S. aureus* were present at higher proportions. This finding is similar to those of other studies from Hawassa and Jimma, Ethiopia [21, 23], and Zimbabwe [30].

In the current study, 171 (95%) of the isolates were resistant to at least one antimicrobial tested, which is similar to a study conducted in Bahir Dar (94.4%) [26]. However, this result was higher than that reported in Debre Markos and Gondar, Ethiopia [18, 27]. This may be due to increased overuse of antibiotics and self-medication, resulting in antibiotic resistance. Generally, amplified resistance is caused by the use of broad-spectrum agents, and the use of medications as a result of insufficient doses or incomplete treatment courses plays a significant role in the emergence of antimicrobial resistance [31].

In this study, the overall multi-drug resistance profile of isolates was 79.4%, which was comparable with findings from Debre Markos, Ethiopia (76.1%) [32]. However, this result was higher compared to studies from Indonesia (47.7%) [25] and Bahir Dar, Ethiopia (61.8%) [26]. This indicates that antibiotic resistance, especially MDR, is a growing risk in Ethiopia. This might be linked to the misuse, overuse, and inappropriate use of antibacterial agents. Hospitals receive referrals from many districts and distant rural villages. These patients received different antibiotic treatments from the referring centers or over-the-counter centers, usually with inappropriate doses, before arriving at the hospital.

Among the gram-negative isolates, the levels of MDR for *K. pneumoniae* and *E. coli* were 90.5% and 90.2%, respectively. The level of MDR for gram-positive bacteria such as *S. aureus* and *S. saprophyticus* were 78.8% and 75%. This finding also showed that gram-negative isolates had a higher rate of MDR compared to other studies, similar to the findings in Bahir Dar [33]. The difference in MDR levels between gram-negative and gram-positive bacteria may be due to different gram-negative mechanisms, including altered target sites, β-lactamase production, decreased antibiotic penetration, and efflux pumps [34].

In this study, sociodemographic and clinical factors were analyzed as independent risk factors for bacterial infection. Using multivariable logistic regression, different variables were identified as contributing factors to bacterial infection. The presence of chronic underlying disease, previous hospitalization, history of invasive procedures, and consumption of raw animal products showed a statistically significant association with the acquisition of bacterial infection.

According to our study, patients with chronic underlying disease had 1.83 times the odds of increased acquisition of bacteria than patients who did not have an underlying disease (95% CI = 1.05–3.17). This might be because participants with underlying chronic diseases may be exposed to many drugs, frequently interact with health professionals, and have a poor immune status, which can contribute to bacterial infection. This finding is supported by those of other studies conducted in Ethiopia [35, 36] and China [37].

The other independent factor associated with bacterial infection was a history of hospitalization. Having a history of previous hospitalization had 1.96 times the odds of increased acquisition of bacteria than patients with no history of previous hospitalization (95% CI = 1.09–3.54). This finding is supported by other studies conducted in Brazil and India [38, 39].

The educational status of the illiterate individuals and those who read and write was also significantly associated with the acquisition of bacteria. Participants with no formal education lacked personal hygiene knowledge. This is similar with studies in Ethiopia [40, 41].

Another factor associated with the acquisition of bacterial infections was the history of invasive procedures. In this study, participants who had a history of an invasive procedure had 2.09 times the odds of acquiring bacterial infection than those who didn’t have such a history (95% CI = 1.06–4.49). This finding is supported by other studies conducted in Ethiopia [42].

## Conclusions and recommendation

The magnitude of community-acquired bacterial infection was 47%. Gram-negative pathogens were predominant that account for more than half of the cases. *E. Coli* and *S. aureus* were the most common pathogens identified. Antimicrobial resistance among common isolates was high for routinely used antibiotics and the rate of MDR was alarming. Chronic underlying disease, previous hospitalization, history of invasive procedures, consumption of raw animal products, and educational status of illiterate, and read and write had significant association were acquisition of bacterial infection. Strengthening of antimicrobial resistance surveillance system, antibiotic stewardship programs and effective antibiotic policy is required to halt the transmission of disease including drug resistant bacteria in the community.

## Data Availability

The findings of this study were generated from the data collected and analyzed based on the stated methods and materials. All data have already been found in the manuscript, and there are no supplementary files. The original data supporting this finding are available upon request.

## Declarations

We declare that this manuscript is original and has not been presented or published in this University or institution. And also, all methods were achieved based on appropriate guidelines and regulations.

## Abbreviations

AIDS: Acquired Immune Deficiency Virus
AMR: Anti-Microbial Resistance
BSI: Blood Stream Infection
CLED: Cysteine Lactose Electrolyte Deficient
CLSI: Clinical and Laboratory Standard Institute
CoNS: Coagulase Negative Staphylococcus
CSF: Cerebrospinal Fluid
HIV: Human Immune Deficiency Virus
ICU: Intensive Care Unit
LRTI: Lower Respiratory Tract Infection
MDR: Multi Drug Resistant
MRSA: Methicillin-Resistant Staphylococcus Aureus
MR-VP: Methyl Red-Vogues Proskauer
NICU: Neonatal Intensive Care Unit
TSI: Triple Sugar Iron
UTI: Urinary Tract Infection
WHO: World Health Organization
XLD: Xylose Lysine Decarboxylate
